# Does the Difference in Axial Length Affect the Refractive Outcome?

**DOI:** 10.21315/mjms2024.31.1.6

**Published:** 2024-02-28

**Authors:** Noor Amalina Saidi, Nur Syahirah Abdul Karim, Adawiyah Ismail, Raja Nor Farahiyah Raja Othman, Nor Higrayati Ahmad Kasah, Azhany Yaakub, Qi Zhe Ngoo

**Affiliations:** 1 Department of Ophthalmology and Visual Science, School of Medical Sciences, Universiti Sains Malaysia, Kelantan, Malaysia; 2 Department of Ophthalmology Hospital Sultanah Nur Zahirah, Terengganu, Malaysia

**Keywords:** axial length, cataract surgery, refractive outcomes, spherical equivalent, SRK2

## Abstract

**Background:**

The purpose of this study is to compare axial length (AL) and the refractive outcome after phacoemulsification surgery from 2014 to 2019 at Hospital Sultanah Nur Zahirah, Terengganu, Malaysia.

**Method:**

This was a retrospective record review of all cataract patients who met the inclusion criteria and underwent uneventful superior wound phacoemulsification with nontoric intraocular lens (IOL) by a single surgeon from 2014 to 2019. Using optical biometry or immersion technique, the preoperative AL determined solely via the Sanders, Retzlaff and Kraff 2 (SRK2) formula was selected. The postoperative spherical equivalent (SE) at 6 weeks–12 weeks was retrieved. Using Statistical Package for the Social Sciences version 24.0, the mean differences between targeted and actual postoperative SE were analysed based on the AL.

**Result:**

In this study, 490 eyes of 472 patients aged 25 years old–88 years old (mean age 65.72 years old [SD 8.83]) were involved. There were 162 eyes (33%) in Group A (< 23 mm), 189 eyes (39%) in Group B (23.01 mm–24.0 mm) and 139 eyes (28%) in Group C (> 24.0 mm). The mean AL was 23.63 mm (SD 1.19). The mean differences between the targeted and actual postoperative SE were: −0.09 D (SD 0.60) in Group A, −0.07 D (SD 0.53) in Group B and −0.16 D (SD 0.52) in Group C. No significant difference was found between these groups (*P =* 0.327).

**Conclusion:**

There was no significant difference in the refractive outcome using the SRK2 formula in different ALs after phacoemulsification surgery. Hence, there is no reason to modify or adjust the targeted SE based on AL.

## Introduction

Achieving the target refraction postoperatively is the most crucial aspect of cataract surgery. Patient satisfaction following modern cataract surgery requires good surgical skills and increasingly superior refractive outcomes. Patients and doctors often desire to achieve emmetropia after cataract surgery. Despite ongoing advancements in preoperative and intraoperative diagnostics, refractive planning, and surgical technologies, the residual refractive error continues to be a major source of post-cataract surgery dissatisfaction. Improving refractive outcomes and correcting residual astigmatic or spherical refractive defects postoperatively becomes crucial to meeting patients’ surgical result expectations (1). Several factors may contribute to the postoperative refractive outcomes, for example, the preoperative biometry measurements. The axial length (AL) measurement is part of the biometry used to determine intraocular lens (IOL) power. The AL should be accurately measured in order to achieve good visual outcomes from cataract surgery (2). Residual astigmatism and refractive error will compromise the patient’s vision and satisfaction (3). This study aims to determine whether the AL factor will affect the postoperative target refraction, as eyes with ALs shorter or longer than the normal range have classically had a high rate of refractive error after cataract surgery.

## Methods

### Patient Selection

All cataract patients involved in this study met the inclusion criteria, including those who underwent uneventful superior wound phacoemulsification with the non-toric IOL under local or general anesthesia by a single surgeon at the Hospital Sultanah Nur Zahirah, Terengganu, Malaysia from 2014 to 2019. Additionally, using optical biometry or immersion technique, the preoperative AL determined solely using the Sanders, Retzlaff and Kraff 2 (SRK2) formula was selected. The exclusion criteria include complicated cataract surgery, sutured wound, multifocal or toric IOL, AL measurements other than the SRK2 formula and biometry done using the contact method.

### Data Gathering

This study involved a retrospective record review of all postoperative refractions at 6 weeks–12 weeks. The data were retrieved from the National Cataract Surgery Registry website. First, the spherical equivalent (SE) was calculated and then, the mean differences between targeted SE and actual postoperative SE were recorded.

### Data Analysis

The data obtained were subsequently analysed based on the AL. One-way analysis of variance (ANOVA) was employed to compare the means of three independent groups based on the AL using the Statistical Package for the Social Sciences version 24.0.

## Results

**Figure 1:**
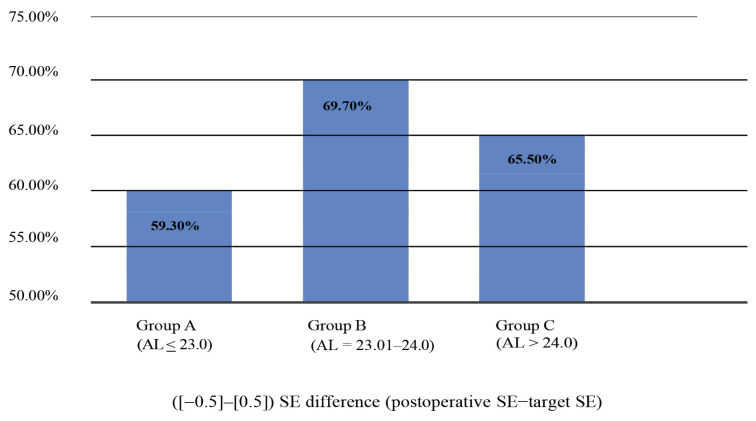
Postoperative SE achieved using different ALs in Groups A, B and C Notes: *SE = spherical equivalent; AL = axial length

In this study, 490 eyes of 472 patients aged 25 years old–88 years old (mean age 65.72 years old [SD 8.83]) were involved. There were 162 eyes (33%) in Group A with AL (< 23 mm), 189 eyes (39%) in Group B with AL (23.01 mm–24.0 mm) and 139 eyes (28%) in Group C with AL (> 24.0 mm). The mean AL was 23.63 mm (SD 1.19). As shown in Figure 1, 59.30% in Group A achieved postoperative SE within (−0.5)–(0.5), 69.7% in Group B and 65.50% in Group C.

Mean differences between the targeted SE and actual postoperative SE were −0.09 D (SD 0.60) in Group A, −0.07 D (SD 0.53) in Group B and −0.16 D (SD 0.52) in Group C, respectively. The results concluded no significant difference between these groups (*P* = 0.327) (Table 1).

## Discussion

Cataract surgery is an essential ophthalmic procedure considering that cataract is the leading cause of preventable blindness worldwide, which accounts for 45%–60% of the cases of blindness in the developing world (4). In this modern era of cataract surgery, the surgery aims to provide patients with an exceptional postoperative refractive outcome. Many factors affecting the surgical outcomes must be considered, including patient characteristics such as age, underlying systemic and ocular comorbidities, biometry techniques and measurements, and intraoperative complications (5).

Refractive error after cataract surgery may result from calculation errors where the IOL power calculation errors can lead to a significant refractive error after cataract surgery. The factors that contribute to these errors include incorrect AL or corneal power measurements and improper selection of the IOL power formula (6).

Ocular parameters for IOL calculation can be measured using a variety of approaches, including optical biometry, immersion or contact technique. Optical biometry is the new standard for IOL calculation since it is a non-contact technique that eliminates the risk of patient cross-contamination is avoided and the need for topical anaesthesia (7). Additionally, unlike ultrasound biometry, optical biometry includes keratometry; hence, no extra instruments are required to determine IOL power. However, the optical biometry method requires an adequate foveal fixation for optimal alignment; in cases of poor visual acuity of less than 20/200 where patients cannot fixate the eye, the ultrasound method is preferred (8).

There has not been enough research done to determine whether the AL difference affects the refractive outcome of cataract surgery. According to Fotedar et al. (9), AL alterations have the most significant potential association with SE refraction change. An AL difference of only 0.2 mm is linked to a greater likelihood of refractive errors exceeding 0.5D from the target value. A study by Karabela et al. (10) showed a slight hyperopic shift in medium-size AL, 22.0–24.60 mm. Another study by Jun and Lee (11) showed that hyperopic shift tends to occur in short AL. These data contradicted our study, which showed slight myopic shifts in all groups of AL with no significant difference between the groups. Previous studies have also shown that myopic patients in their series were more likely to demonstrate a postoperative refractive error (only 70.7% of myopic patients versus 82.1% of nonmyopic patients were within 0.5 D of predicted refraction) (12, 13). Our study showed that more than 50% were within 0.5 D difference from the targeted SE in all groups of AL.

Expectations for refractive outcomes have heightened as cataract surgery has progressed into lens-based refractive surgery. Over the last decade, technological breakthroughs have enabled new methods for measuring the cornea in preparation for cataract surgery. With the increasing capacity to correctly quantify corneal power, each patient’s most precise IOL may be determined. Moreover, new technology measures the anterior and posterior corneal surfaces to evaluate corneal power and aberrations more accurately. These measures assist surgeons in making the most informed judgments about the power of the IOL to be placed during cataract surgery (14).

Another factor that can affect the postoperative outcome is astigmatism. Astigmatism is a common pre-existing condition that can affect the accuracy of IOL power calculations and can also cause a significant refractive error after cataract surgery if not adequately addressed (15). Inoue et al. (16) concluded that throughout the 20-year follow-up period following small-incision cataract surgery, postoperative astigmatism continued to move toward against the rule (ATR) astigmatism, which appears to mirror the normal course of corneal astigmatic changes that occur with aging. Regardless of the kind of preoperative astigmatism, ATR astigmatic changes were identical in pattern and amplitude. Corneal astigmatism was substantially increased following a long clear corneal incision compared to a short clear corneal incision, and the wound-related form alterations started shortly after surgery but quickly faded (17).

To minimise refractive error after cataract surgery, surgeons employ various techniques, including advanced IOL calculation formulas, toric IOLs for astigmatism correction and femtosecond laser-assisted cataract surgery. Furthermore, patients may benefit from a detailed preoperative evaluation to identify any pre-existing refractive errors and discuss treatment alternatives.

## Conclusion

The AL measurement is part of the biometry used to determine the power of an IOL. Consequently, accurate measurement of the AL is necessary for optimal visual results after cataract surgery. At varied ALs, our investigation found no significant differences in the refractive outcomes using the SRK2 formula. Therefore, there is no reason to modify or adapt the planned SE based on AL.

## Figures and Tables

**Table 1:** No significant difference between the different ALs in Groups A, B and C with the postoperative SE

Group	AL (mm)	Actual post-op SE, mean D (SD)	Targeted SE, mean D (SD)	Different (post-op-target), mean D (SD)	*P*-value
A	< 23	−0.59 (0.58)	−0.50 (0.12)	−0.09 (0.60)	*P* = 0.327 (One-way ANOVA)
B	23.01–24.0	−0.56 (0.52)	−0.49 (0.12)	−0.07 (0.53)	
C	> 24.0	−0.73 (0.53)	−0.57 (0.20)	−0.16 (0.52)	

Notes:

* SE = spherical equivalent; AL = axial length
